# A Hybrid Photoplethysmography (PPG) Sensor System Design for Heart Rate Monitoring

**DOI:** 10.3390/s24237634

**Published:** 2024-11-29

**Authors:** Farjana Akter Jhuma, Kentaro Harada, Muhamad Affiq Bin Misran, Hin-Wai Mo, Hiroshi Fujimoto, Reiji Hattori

**Affiliations:** 1 Major of Device Science and Engineering, Interdisciplinary Graduate School of Engineering Sciences, Kyushu University, Fukuoka 8160811, Japan; jhuma.farjana.akter.233@s.kyushu-u.ac.jp (F.A.J.);; 2 OPERA Solutions Inc., 5-5 Kyudai-Shimmachi, Nishi-ku, Fukuoka 8190388, Japan

**Keywords:** heart rate, light-emitting diode (LED), LED duty cycle, LED luminous intensity, organic photodetector (OPD), photoplethysmography (PPG)

## Abstract

A photoplethysmography (PPG) sensor is a cost-effective and efficacious way of measuring health conditions such as heart rate, oxygen saturation, and respiration rate. In this work, we present a hybrid PPG sensor system working in a reflective mode with an optoelectronic module, i.e., the combination of an inorganic light-emitting diode (LED) and a circular-shaped organic photodetector (OPD) surrounding the LED for efficient light harvest followed by the proper driving circuit for accurate PPG signal acquisition. The performance of the hybrid sensor system was confirmed by the heart rate detection process from the PPG using fast Fourier transform analysis. The PPG signal obtained with a 50% LED duty cycle and 250 Hz sampling rate resulted in accurate heart rate monitoring with an acceptable range of error. The effects of the LED duty cycle and the LED luminous intensity were found to be crucial to the heart rate accuracy and to the power consumption, i.e., indispensable factors for the hybrid sensor.

## 1. Introduction

**Figure 1:**
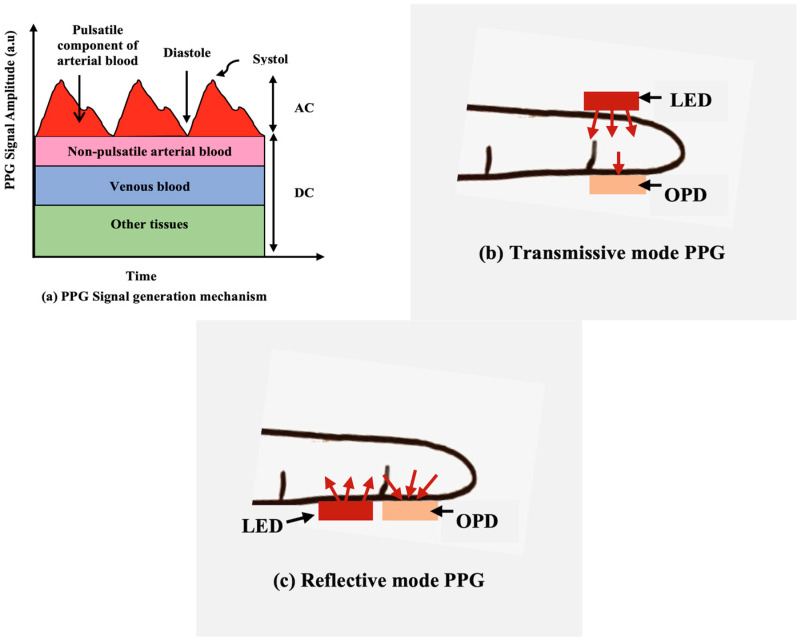
(**a**) Schematic representation of the amplitude over time to explain PPG signal generation through tissues. (**b**) Schematic illustrations of transmissive mode PPG (LED and OPD are placed on the opposite side of the sensing location). (**c**) Schematic illustrations of reflective mode PPG (both LED and OPD are placed side by side on the sensing location).

Photoplethysmography (PPG) is a simple, cost-effective, and non-invasive optical sensing technique for measuring the blood volume changes through the arteries during each cardiovascular pulsation cycle. The low cost and convenient wearability of PPG sensors have made them applicable to monitoring basic health condition signs like heart rate, pulse, respiration rate, oxygen saturation, and heart rate variability [1,2,3]. A PPG sensor uses a light receiver (photodetector, PD) to detect the reflected or transmitted light from human skin. The variation in the signal intensity is associated with the volumetric changes in blood after the skin is illuminated with a light-emitting source, i.e., a light-emitting diode (LED) [4]. Figure 1a shows the basic mechanism of PPG signal generation in body tissues. According to the relative position of the LED and the PD, a PPG sensor works either in transmissive (placement of LED and PD on the opposite side) or reflective mode (placement of both LED and PD on the same side) to obtain the PPG signal. Conventional transmissive mode PPG sensors employ a combination of solid-state LED and a PD to acquire the PPG signal by detecting the penetrated light through body parts, such as fingertips and earlobes, but it suffers from a low signal-to-noise ratio and limitation of sensing circumstances [5,6]. In contrast, in the case of the reflective mode PPG sensor, the LED and the PD can be placed close to each other in any part of the body to obtain the signal, e.g., through the skin without deep light penetration, thus facilitating user-friendly monitoring [7,8]. The acquisition method of PPG in both transmissive and reflective modes is shown in Figure 1b,c.

Though conventional PPG sensors with silicon and other inorganic PDs can offer high carrier mobility, a tunable bandgap, and a low exciton binding energy to enable high detectivity, they still confront some issues, e.g., the expensive and complicated fabrication process, the data inaccuracy due to fragility and roughness of human skin and the limitation perception depending on the body part [6,9,10]. To overcome these challenges, organic optoelectronic devices (organic light-emitting diode, OLED, and organic photodetector, OPD) have been adopted in the studies of many researchers in the last decade because of their flexibility, light weight, cost-effectiveness, and large-area production capabilities [10,11,12,13]. The design flexibility of optoelectronic devices allows various types of structural combinations of OPDs and OLEDs, enabling an efficient PPG signal acquisition from the skin interface with lower power consumption. For example, an all-organic transmissive mode PPG sensor with two rectangular-shaped OPDs and two green and red OLEDs was demonstrated, and it provided the pulse rate and oxygenation level with a low percentage of error comparable to that of inorganic devices [5]. A monolithic PPG array with square-shaped OLEDs and OPDs was proposed for measuring oxygen saturation [6]. A reflective patch-type PPG sensor with a combination of flexible circular OLEDs and a wrap-around OPD was capable of detecting reliable signals of heart rate and oxygen saturation from various body parts with ultra-low power consumption [14]. A reflective mode PPG sensor was reported with varying geometrical conditions and with the introduction of an optical barrier between the light source and detector to reduce the light scattering [15]. Another all-organic pulse oximeter based on the PPG principle was demonstrated by Fahed et al. with a red LED and OPD combination that was able to obtain PPG signals from different positions of the human body with a 5% improvement in the signal-to-noise ratio of the system [16].

Although such all-organic PPG sensors have the advantage of lower power consumption for continuous health monitoring, they suffer from a short lifetime of OLED operation for practical use. Furthermore, OLEDs used in such organic sensors with small device areas operated at relatively high brightness to obtain an intense signal can be subjected to fast degradation [14]. These problems can be overcome with a hybrid sensor configuration, i.e., a combination of inorganic LEDs and an organic photodetector, OPD. Very few researchers have explored the possibilities of using an inorganic light emitter–organic light detector combination for PPG sensors that can monitor health conditions mostly using NIR illumination. Park et al. proposed an ultra-flexible near-infrared (IR)-responsive skin-conformal PPG sensor with a combination of a PIPCP polymer acceptor–PC_61_BM donor-based OPD and a commercially available IR LED to measure the blood pressure applicable on a human fingertip [17]. Lee et al. demonstrated a hybrid reflective sensor with an inorganic red and NIR LED and a wrap-around OPD via lamination with structural optimization by optical simulation [18]. They adopted a bulk-heterojunction-type active layer based on the non-fullerene-based polymer acceptor 2,2′-[[4,4,9,9-tetrakis(4-hexylphenyl)-4,9-dihydro-s-indaceno[1,2-b:5,6-b′]dithiophene-2,7-diyl]bis[[4-[(2-ethylhexyl)oxy]-5,2-thiophenediyl]methylidyne (5,6-difluoro-3-oxo-1*H*-indene-2,1(3*H*)-diylidene)]] bis[propanedinitrile] (IEICO-4F) and poly([2,6′-4,8-di(5-ethylhexylthienyl) benzo [1,2-b;3,3-b]dithiophene]{3-fluoro-2[(2-ethylhexyl) carbonyl] thieno[3,4-b]thiophenediyl}) (PTB7-Th) as a donor that can detect the red and NIR illumination used while measuring PPG signals. In another work, a flexible organic–inorganic hybrid NIR PPG sensor was proposed by Xu et al. with an organic phototransistor and an NIR LED for continuous monitoring of heart rate variability and pulse pressure variation with low power consumption [19].

Considering the limited studies in the case of an inorganic light emitter–organic photodetector for the case of a PPG sensor, in this work, we propose a reflective mode hybrid PPG sensor featuring an inorganic RGB LED surrounded by a circular-shaped organic photodetector (OPD) based on a classic P3HT/PC_61_BM bulk heterojunction active layer with high detectivity in the visible region. The use of the classic P3HT/PC_61_BM OPD enables an easy and cost-effective fabrication process, simplifying the selection of an appropriate organic active layer for light detection within the desired range. The typical P3HT/PC_61_BM-based OPD allows the use of red and green illumination with almost the same penetration depth as IR illumination, which can exhibit better performance and a higher correlation for health condition monitoring both at room temperature and temperatures below 15 °C [20]. The OPD design was optimized by varying the distance between the light source and the detector to enhance performance. A detailed design of the hybrid sensor system is presented, including the methodology for PPG signal acquisition. For sensor validation, the heart rate was calculated from the acquired PPG data. Additionally, to further improve sensor performance, a systematic study was conducted on heart rate accuracy and power consumption under different LED duty cycles and light intensities, aiming to develop a low-power hybrid PPG sensor.

## 2. Instrumental Configuration of the Hybrid Sensor

### 2.1. Overview of Hybrid PPG Sensor System

**Figure 2:**
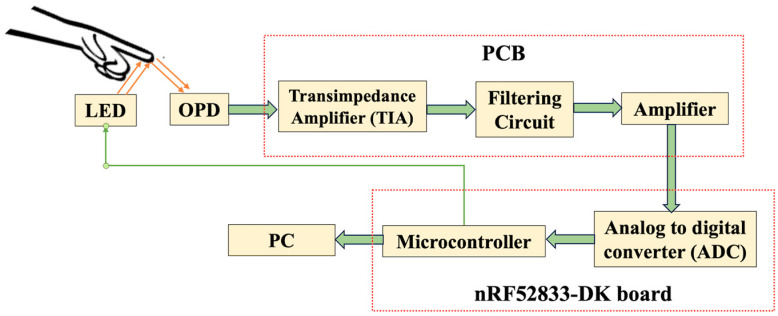
Block diagram of the proposed hybrid PPG sensor system.

Figure 2 shows the block diagram of the proposed hybrid PPG sensor system adopting the reflective mode PPG scheme. The system has an optoelectronic device module containing an OPD as the light detector and an LED as the light emitter mounted on a customized printed circuit board (PCB). The other module holds the PPG driving circuit, the device holder, and the microcontroller unit (MCU). The PPG driving circuit contains a transimpedance amplifier, a filtering circuit followed by an amplifier circuit to modify the obtained PPG signal to an acceptable range by the analog to digital converter (ADC) of the microcontroller (MCU) unit. The modules of the hybrid PPG sensor will be explained in the following sections.

#### 2.1.1. Light Detector and Emitter

**Figure 3:**
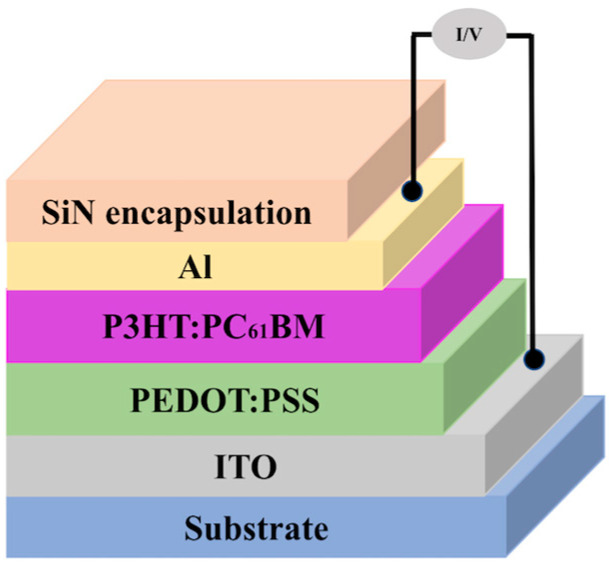
The layer structure of the OPD device.

The light emitter, LED, is a commercially available surface mount device (SMD) type (LED RGB CLR 1206 SMD BOTTOM ENT, Wurth Elektronik, USA) mounted on the customized PCB. The RGB LED has an emission wavelength of 624 nm, 525 nm, and 470 nm, power dissipation of 60 mW, 90 mW, and 90 mW, and spectral bandwidth of 20 nm, 30 nm, and 20 nm for red, green, and blue emissions, respectively. Since the OPD and the LED are on different platforms, to fabricate a hybrid sensor system, it is advantageous to allow the OPD more design flexibility while the commercially available LED has less flexibility in customizing. As shown in Figure 3, the OPD was prepared on a separate substrate from the LED on the PCB, allowing the spin coating of the active and buffer layers of the OPD. The patterning of the OPD through lithography and a metal mask was also performed separately from the LED module.

As the light detector module, a polymeric bulk heterojunction OPD was adopted for the detection of visible light. The layer structure of the OPD is also shown in Figure 3. For fabrication of the OPD, a pre-ITO-coated glass substrate (2.5 cm × 2.5 cm) was properly cleaned and subjected to lithography, followed by a wet etching process for the ITO patterning. The patterned ITO-coated glass substrate was then cleansed with detergent, deionized water, acetone, and isopropyl alcohol (IPA) several times via ultrasonic bathing and then subjected to UV/ozone treatment to remove any residue. Filtered poly(3,4-ethylenedioxythiophene)–poly(styrene sulfonate) (PEDOT/PSS) (Clevious P VP AI 4083, Ossila Limited, Sheffield, UK) solution was then spin-coated on the patterned ITO substrate as the hole transport layer for 60 s at 3000 rpm speed and annealed for 10 min at 200 °C in the air. Subsequently, a blend of poly(3-hexylthiophene-2,5-diyl) (P3HT) (FUJIFILM Wako Chemicals, Tokyo, Japan) and [6,6]-phenyl C_61_-butyric acid methyl ester (PC_61_BM) (Sigma Aldrich, St. Louis, MO, USA) dissolved in chlorobenzene in a 1.8:1 ratio was deposited over the PEDOT/PSS via spin coating at 1000 rpm speed for 50 s as the active layer for photon absorption. Afterward, the device was annealed at 80 °C for 5 min in a N_2_ environment before the vacuum thermal deposition of the Al cathode. Finally, the device was encapsulated with plasma-enhanced chemical vapor deposition (PECVD) of a silicon nitride (SiN) thin-film layer. PECVD-deposited SiN has better moisture permeability and higher electrical stability, which helps the OPD to be less affected by environmental degradation, improving the lifetime of the OPD, i.e., the hybrid sensor [21].

The OPD was electrically connected to the PCB via two spring header pins to facilitate integration with the analog driving circuit for signal processing after the device fabrication.

#### 2.1.2. Analog Driving Circuit and MCU Unit

**Figure 4:**
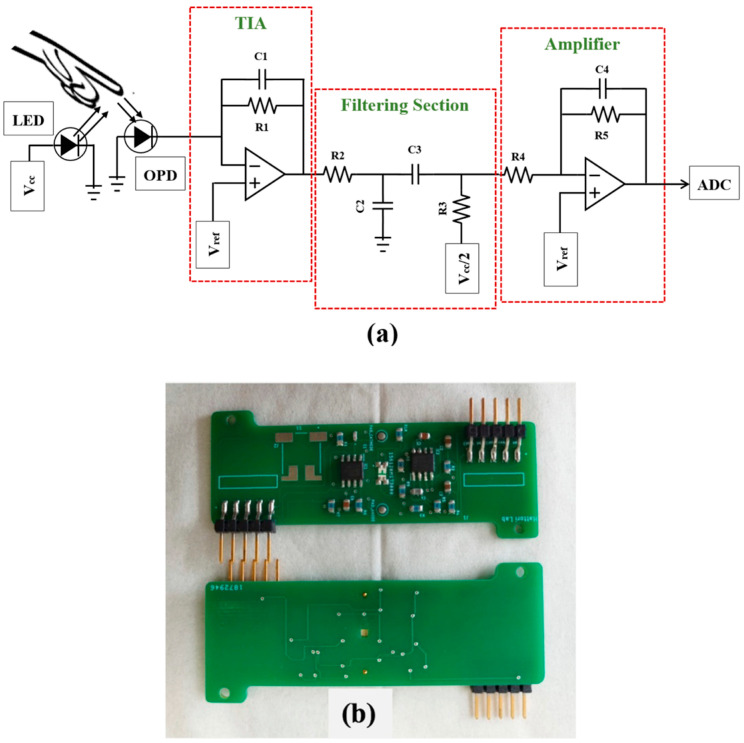
(**a**) Block diagram of the driving circuit containing TIA, filter, and amplifier. (**b**) Customized PCB including the driving circuit and LED.

The analog driving circuit of the sensor system mainly has three stages—the transimpedance amplifier (TIA) circuit, the bandpass filter for noise reduction, and the amplifier circuit for further amplification. As a response to the light illumination onto the skin from the LED, a portion of the light is reflected from the blood vessel and harvested by the photodetector which is very low in amplitude (in the microampere range). At the first stage of the TIA, the current output of the OPD goes through a current-to-voltage converter to translate the current output to a voltage signal. To avoid the impedance mismatch, the TIA circuit offers a low input impedance to the OPD input and insulates it from the operation amplifier voltage output [22,23]. The output of the TIA circuit then passes through the bandpass filtering circuit to remove the high-frequency noise from the ambient light fixture and the alternating current (AC) devices with the low-pass filter portion and eliminate the direct current (DC) signal with the high-pass filter portion. The signal is then applied to the final amplifier for further amplification to a suitable range for the input via universal asynchronous receiver transmitter (UART) communication to the analog-to-digital converter (ADC). The block diagram of the driving circuit is shown in Figure 4a, while the customized PCB board with the driving circuit and LED is displayed in Figure 4b.

The MCU module used in this work was the onboard interfaced MCU included in the nRF52833DK hardware development platform, which supports UART, Bluetooth low energy (BLE), and other communication protocols. The signal through the amplifying stage of the driving circuit was connected to the ADC pin of the nRF52833DK board. SEGGER J-Link OB interface firmware was adopted for programming and debugging the nRF52833 system on chip (SoC) to control the ADC and the LED operation.

#### 2.1.3. Device Holder Design

**Figure 5:**
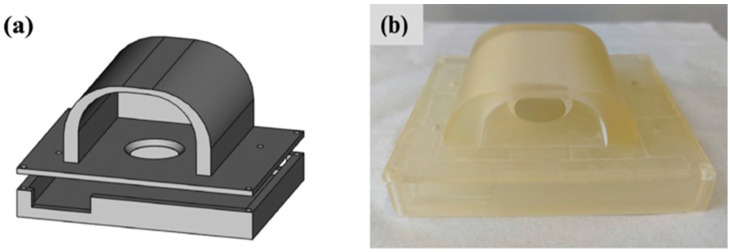
(**a**) FreeCAD design of the device holder. (**b**) Image of the 3D-printed device holder.

A device holder was designed to hold the PCB and the glass substrate containing the OPD together to obtain the PPG signals easily and reproducibly. The FreeCAD design of the device holder is shown in Figure 5a, whereas the image of the device holder is shown in Figure 5b. The structure was prepared using a 3D printer. The holder is composed of an oval-shaped lid intimately in contact with the upper surface of the device to reduce the light interference coming from the ambient light during the signal measurement.

**Figure 6:**
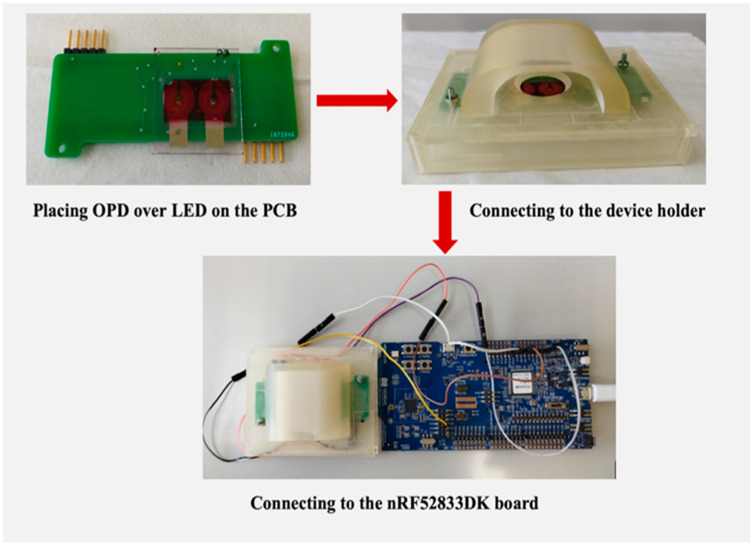
The steps of arranging the PPG sensor system.

The step-by-step process of arranging all the components for PPG detection has been summarized in Figure 6. First, the fabricated OPD on the glass substrate is attached to the PCB board by maintaining the accurate position for light signal harvest. Next, they are fit into the device holder and then connected to the nRF52833DK board, which consists of the ADC and MCU (see also Figure 2), for the further process of PPG signal detection.

## 3. Results and Discussion

### 3.1. OPD Optimization

**Figure 7:**
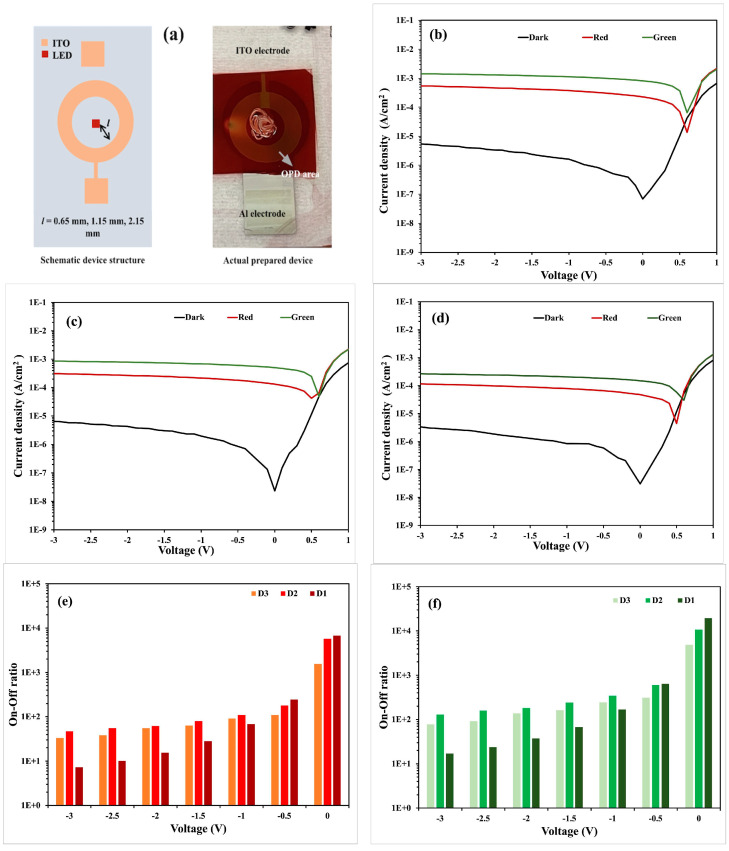
(**a**) Schematic representation image of the OPD and actual image of prepared D1 device. (**b**–**d**) J–V characteristics of the prepared OPD devices D1, D2, and D3, respectively. The black and red solid lines indicate the dark and photocurrent densities, respectively. The photocurrent was obtained under a red LED illumination with a peak wavelength of 624 nm and a luminous intensity of 150.06 mcd. (**e**,**f**) On/off ratio of the OPD at different biasing voltages under the above-mentioned red and green LED illumination conditions.

Affiq et al. showed the importance of keeping the emitter–detector distance lower than 3 mm in the case of reflectance mode PPG operation for achieving high photocurrent, minimizing the loss of light, and reducing the disturbance of the optical path through the OPD [24]. OPD devices exhibited a high dark current and low photocurrent density, leading to a reduced on/off ratio—a critical parameter for reflective mode PPG sensors, particularly as the LED-OPD distance increases [25]. In this study, three different OPD device geometries were chosen based on the emitter–detector distance for optimizing the OPD structure of the hybrid PPG sensor. A circular shape surrounded the LED to harvest the light reflecting from all directions efficiently. Figure 7a shows the schematic illustration of the OPD structures, where *l* denotes the emitter–detector distance (chosen arbitrarily as 0.65 mm (D1 device), 1.65 mm (D2 device), 2.15 mm (D3 device)), and image of the actual prepared D1 device (D2 and D3 have similar structures with only variation in *l*). The J–V characteristics curves for the three devices D1, D2, and D3 are displayed in Figure 7b–d with embedded actual structures, respectively.

The exhibited dark current (current flowing through the OPD with no light input) and photocurrent (current flowing through the OPD with incident light input) density of the OPDs for red LED illumination (peak wavelength = 624 nm, luminous intensity = 150.06 mcd) and green LED illumination (peak wavelength = 525 nm, luminous intensity = 7.84 cd) at zero bias condition are summarized in Table 1.

The low dark current and high photocurrent of the D1 device compared to the D2 and D3 devices resulted in a high on/off ratio (ratio of photocurrent and dark current, 3–4 orders of magnitude), which allowed us to choose the D1 device for further hybrid sensor analysis. The on/off ratio comparison graphs for all three OPD devices for red and green illumination are depicted in Figure 7e,f. Although the OPD demonstrated good photoresponse under both red and green illumination, this study utilizes only red illumination for the acquisition of the PPG signal, which suffices for heart rate calculation and sensor system validation. Future improvements to the hybrid sensor will explore green illumination for additional functionalities including peripheral oxygen saturation (SpO_2_) and heart rate variability analysis.

### 3.2. PPG Signal Acquisition

**Figure 8:**
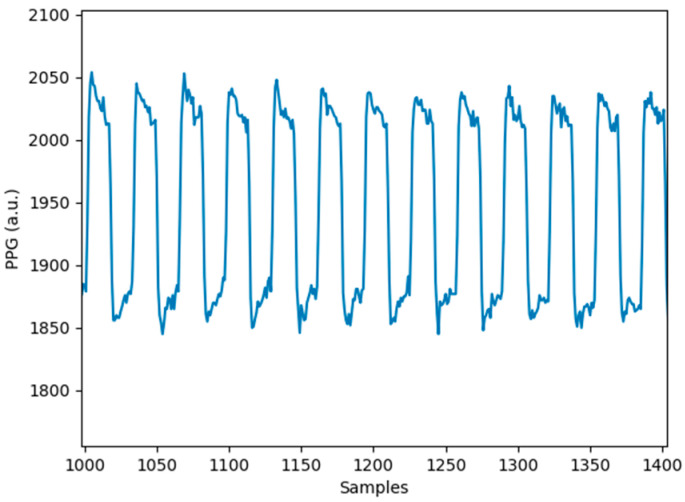
Obtained PPG signal with hybrid PPG sensor.

Consideration of the PPG sampling rate is one of the keys to designing a PPG sensor as a lower sampling rate can reduce the power consumption. However, a low sampling rate often deteriorates the resolution of the PPG signal, leading to inaccurate measurement data [26,27]. With this consideration in mind, most researchers have been employing a sampling frequency of 250 Hz or higher for the detection of accurate PPG for their healthcare product demonstrations [28,29,30]. In this study, we have also sampled the PPG signal at a rate of 250 Hz (4 ms) by the ADC operation. The sampling rate is sufficient for PPG detection as the frequency of the signal ranges between 0.5 and 5 Hz. The LED was sequentially turned on for 64 ms and off for 64 ms during the PPG signal acquisition. The PPG signal obtained from the hybrid PPG sensor is shown in Figure 8.

Typically, a PPG signal shows two main peaks within one cardiac cycle: the first and larger peak, associated with the systolic phase, and the second, smaller peak, corresponding to the diastolic phase. However, the observability of the second peak can be affected by factors like the close positioning of the two peaks, a smaller amplitude of the second peak, and high contact pressure during measurement, temperature, and sensor configuration [31,32]. In this study, the second peak is not prominently visible in Figure 8, likely due to the applied pressure during measurement. To enhance the detection and visibility of the second peak, symmetrical curve fitting, Gaussian curve fitting, and other advanced signal processing techniques can be employed. These methods can help reduce measurement artifacts and improve peak identification, crucial for accurately assessing health conditions like blood pressure and blood glucose levels. Future studies will explore these signal processing approaches to enhance the reliability of the PPG signal, particularly in identifying the diastolic peak, which plays a significant role in health monitoring applications.

### 3.3. Heart Rate Estimation

**Figure 9:**
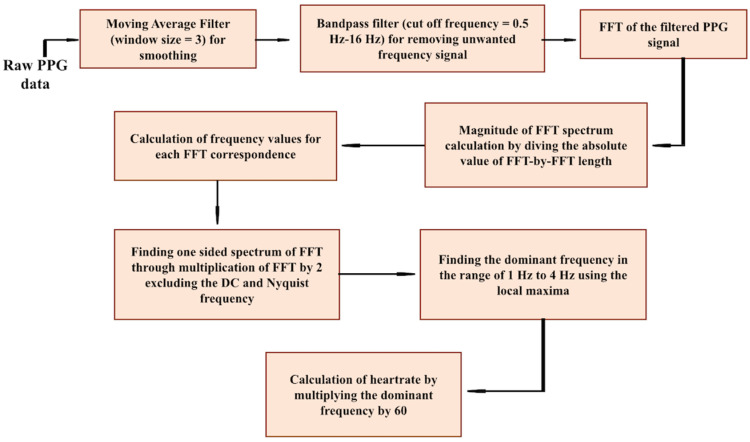
Process flow chart of heart rate estimation.

The process of heart rate estimation in this work is summarized in Figure 9. For the portable use of a PPG sensor, the signal is susceptible to noise corruption and motion artifacts, which are difficult to avoid by using analog filters. Several methods of digital signal processing for noise reduction such as discrete wavelet transform, cluster analysis, and finite impulse response (FIR) filtering have been applied in many studies. In this work, we employed a combination of moving average and Butterworth bandpass filters to remove noise-induced effects during signal detection from the fingertip. The PPG signal was first smoothed by passing through a moving average filter defined by the following equation:

(1) $$y\left[n\right]=\frac{1}{L}\;\sum_{k=0}^{L-1}\left(x\left[n-k\right]\right),\;n=L,\;L+1,\;\ldots ,M,$$

where *y*[*n*] is the output signal, *L* is the window size, and *M* is the data length. For this work, a moving average filter with window size 3 was chosen to first smooth the signal, which was then passed through a sixth-order Butterworth bandpass filter with a cut-off frequency of 0.5 Hz to 16 Hz for rejecting all noises outside the desired range. Subsequently, a fast Fourier transform (FFT) was executed over the filtered signal for transitioning in the frequency domain. After the computation of the one-sided spectrum of the signal, the peak, i.e., the harmonic in the desired range of frequency (1–4 Hz), was found and multiplied by 60 to obtain the heart rate using the formula [33]

(2) $$Heart\;\;rate\;\left(bpm\right)=60\times Dominant\;Frequency\;of\;PPG\;\left(Hz\right)$$

Although the FFT analysis demonstrated good performance for the limited dataset used in this study, its effectiveness may be compromised when dealing with larger datasets susceptible to high motion artifacts and ambient noise. The robustness of FFT analysis in such conditions will be further investigated in future work by comparing it with complementary approaches, such as wavelet transforms and adaptive filtering techniques, which are better suited for handling noise-affected signals.

**Figure 10:**
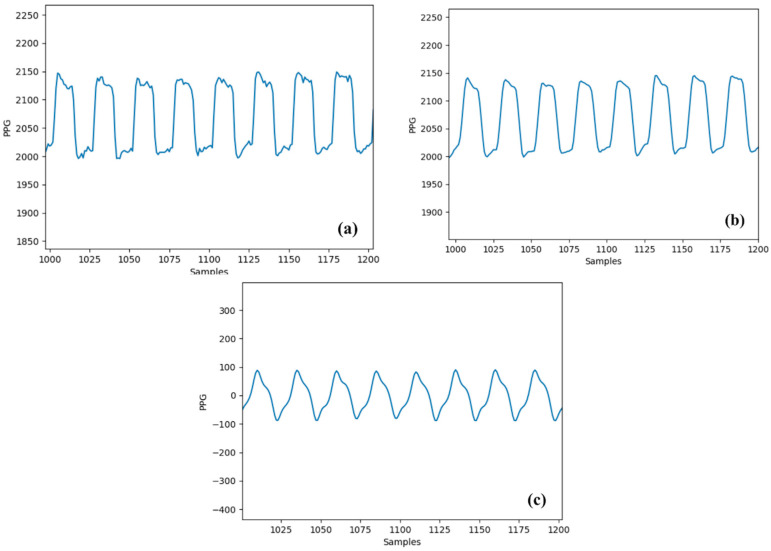
(**a**) PPG signal before filtering (some disturbance can be seen at the signal peak due to the disturbance introduced while measuring from fingertip). (**b**) PPG signal after applying a moving average filter with a window size 3 to smooth the signal. (**c**) PPG signal after moving average and Butterworth bandpass filter with a cut-off frequency of 0.5 Hz–16 Hz to remove unwanted frequency signals.

Using the methodology shown in Figure 9, heart rate was extracted from the collected PPG signal with a sampling frequency of 250 Hz with a 50% duty cycle of the LED illumination for a specific period during a resting condition of the trial subject. The collected signal corrupted by finger motions was filtered by the moving average filter with the window size 3, followed by the Butterworth bandpass filtering. The comparison between the original PPG signal, the signal after applying the moving average filtering, and the signal after the bandpass filtering is shown in Figure 10. The calculated heart rate was compared with a commercially available portable pulse oximeter (NURSE ANGIE, Custom Co., Tokyo, Japan) that can measure the heart rate within the range of 25 bpm to 250 bpm with a 1 bpm resolution. The calculated heart rate from our hybrid sensor showed an error of 3.33% compared to the commercial device. With an assumption about the heart rate of the commercial device being accurate, the error of the measured heart rate with the hybrid PPG sensor showed an error within an acceptable range that was investigated with laboratory-based research previously, which indicated keeping the error below 5% for heart rate calculation [34].

### 3.4. Power Consumption Management

For a portable PPG sensor device, the LED input power and power efficiency, as well as the power efficiencies of driving and readout circuits, are mainly responsible for the total power consumption of the device. As the size of the sensor and battery capacity are limited to a certain level, it is necessary to reduce the power consumption of the sensor. Methods like adopting different sampling schemes and redesigning the TIA circuit were applied previously to reduce the power consumption of the PPG sensor, which can result in some errors and needs complex circuit analysis in some cases [34,35]. In this work, LED duty cycle and brightness control were adopted to control the power consumption of the hybrid PPG sensor, which can easily be controlled by the PWM operation of the used microcontroller unit.

#### 3.4.1. Controlling LED Duty Cycle

LED power consumption depends highly on the duty cycle of an LED, i.e., the percentage of the period during which an LED is in its active state. The duty cycle of an LED can be decreased by an increased pulse repetition interval (PRI). However, decreasing the duty cycle can reduce the accuracy of the heartbeat interval measurement due to a degraded signal-to-noise ratio and sampling errors [36]. To investigate the tradeoff relation, we measured the PPG signals for various duty cycles (20% to 80%) with 128 ms PRI. The heart rate accuracy that was measured from the comparison of the data from the NURSE ANGIE pulse oximeter (operating at 3V with a power consumption of 200 mW) and the hybrid sensor device and the power consumption of the LED concerning the various LED duty cycles are summarized in Table 2.

From Table 2, it is prominent that a higher duty cycle provides more accurate heart rate detection but consumes more power than a lower duty cycle. For PPG signals obtained during the larger duty cycle, features that help to obtain the heart rate more accurately can be extracted from the signal. On the contrary, a larger duty cycle needs more power to keep the LED in the on state. To provide a balance between these two factors, we maintained a 50% duty cycle or all the other measurement conditions with our hybrid PPG sensor.

#### 3.4.2. Controlling LED Brightness

Another way of optimizing the power consumption of the PPG sensor is to control the incident LED brightness, i.e., the luminous intensity of the LED light source. In a general definition, luminous intensity is the measure of the wavelength-weighted power radiated in a specific direction per constituent solid angle. The LED luminous intensity has a proportional relationship to its power consumption, which can be controlled by the driving current applied. To observe the effect of LED luminous intensity on the power consumption and heart rate accuracy, we varied the luminous intensity of the LED. The results of the heart rate variation measured in the same way for the LED duty cycle and the power consumption for different LED luminous intensities are summarized in Table 3.

From Table 3, it can be seen that the power consumption value increases as the luminous intensity, i.e., the brightness of the LED, increases. More photon extraction needs more power to be consumed. On the other hand, it is evident that the heart rate accuracy does not depend on the luminous intensity of the LED, which is within the acceptance range of error even with a low LED power consumption regime. This indicates that, as far as the reflected light harvesting is sufficient for the PPG signal acquisition, the sensor system can be operated with low brightness conditions to reduce power consumption, which in turn can improve the battery lifetime of the sensor.

The data in Table 2 show that increasing the LED duty cycle enhances sensor performance accuracy at the cost of higher power consumption. Conversely, Table 3 indicates that increasing LED brightness significantly raises power consumption without substantially reducing the error percentage. To optimize sensor performance, a tradeoff can be maintained between these parameters. Operating the sensor at lower brightness with a higher duty cycle is recommended to achieve low power consumption while maintaining an acceptable error range for heart rate measurement.

## 4. Conclusions

In conclusion, we have proposed a hybrid reflective mode PPG sensor based on an inorganic LED and a circular-shaped OPD for efficient light detection in the visible spectrum. The combined effects of the long lifetime of the inorganic LED and the low cost, easy pattern ability, and easy fabrication process of the OPD were utilized for making an efficient PPG sensor with a customized driving circuit. The optimized OPD structure based on the emitter–detector distance ensured a high on/off ratio (up to 3–4 orders) at zero bias, which is crucial for PPG signal detection. The easy and inexpensive fabrication process along with the SiN encapsulation increased the repetitive fabrication possibilities with lower environmental degradation, improving the lifetime of the sensor. The PPG signal obtained at a 250 Hz sampling rate passed through a combination of moving average and Butterworth bandpass filtering, effectively removing the noise generated in the signal detection from the fingertip. The heart rate of the hybrid PPG sensor was calculated through FFT analysis, and by comparison with a commercial device, the accuracy of the heart rate was found to be sufficiently reliable.

Further study of the correlation between the various LED duty cycles and the luminous intensities for improving the sensor’s performance revealed the tradeoff between the accuracy of heart rate and the LED power consumption, which is an important aspect of the system design. A high LED duty cycle resulted in a more accurate heart rate. Still, it required more power, which led to maintaining a 50% duty cycle in this work to provide a balanced relation between the heart rate accuracy and the power consumption. The study of various LED brightness levels indicated that an accurate heart rate measurement is possible with lower LED luminous intensities, i.e., with low power consumption, leading to an improvement in the battery lifetime of the sensor. This study demonstrated that the hybrid PPG sensor, incorporating an optimized OPD structure, is a promising solution for low-power heart rate monitoring. By leveraging a controlled LED duty cycle and luminous intensity, the sensor achieves efficient PPG signal acquisition. Additionally, its application using green illumination shows potential for monitoring other health parameters, such as blood oxygen saturation and breath rate, in future developments.

## Figures and Tables

**Table 1:** Dark and photocurrent density for OPD devices at zero bias condition.

Device Name	Dark Current Density (A/cm^2^)	Photocurrent Density (A/cm^2^)	
		Red Illumination	Green Illumination
D1 (l = 0.65 mm)	3.33 × 10^−8^	2.27 × 10^−4^	8.18 × 10^−4^
D2 (l = 1.65 mm)	2.31 × 10^−8^	1.33 × 10^−4^	5.04 × 10^−4^
D3 (l = 2.15 mm)	3.07 × 10^−8^	4.78 × 10^−5^	2.22 × 10^−4^

**Table 2:** Effects on heart rate accuracy and power consumption for varying duty cycles.

Duty Cycle (%)	Heart Rate (bpm) Variation			Power Consumption of LED (mW)
	Heart Rate from NURSE ANGIE (bpm)	Heart Rate from Hybrid PPG Sensor (bpm)	Percentage Error (%)	
20	80	84	5	13.754
30	82	86	4.88	20.631
40	79	82	3.78	27.508
50	90	87	3.33	34.385
60	81	83	2.47	41.262
70	85	83	2.35	48.139
80	86	84	2.32	55.016

**Table 3:** Effects on heart rate accuracy and power consumption for varying LED brightness, i.e., luminous intensity, levels.

Luminous Intensity (mcd)	Heart Rate (bpm) Variation			Power Consumption of LED (mW)
	Heart Rate from NURSE ANGIE (bpm)	Heart Rate from Hybrid PPG Sensor (bpm)	Percentage Error (%)	
7.68	88	85	3.4	3.310
48.64	84	81	3.7	4.496
445.44	86	89	3.37	12.457
609.28	88	85	3.4	16.290
919.04	91	88	3.29	28.577
1090.56	90	87	3.33	34.385

## Data Availability

Data are contained within this article.
